# Ferroptosis inhibition via the ROS-GPX4 axis drives microplastic-induced malignant progression of nasopharyngeal carcinoma

**DOI:** 10.1186/s12967-025-07508-w

**Published:** 2025-12-22

**Authors:** Xiangying Deng, Xinglong Liu, Juan Feng, Lin Zhao

**Affiliations:** 1 Department of Pathology, The Second Xiangya Hospital, Central South University, Changsha, Hunan 41001l China; 2 Hunan Clinical Medical Research Center for Cancer Pathogenic Genes Testing and Diagnosis, Changsha, Human 410011 China; 3 Institute of Medical Sciences, National Clinical Research Center for Geriatric Disorders, Xiangya Hospital, Central South University, Changsha, Hunan 410008 China

**Keywords:** Microplastics, Nasopharyngeal carcinoma, Ferroptosis, ROS, Tumor metastasis

## Abstract

**Background:**

Microplastics (MPs), as emerging environmental pollutants, have been closely linked to cancer development and progression. However, their specific role in nasopharyngeal carcinoma (NPC) remains unclear. This study aimed to investigate the potential mechanisms by which polystyrene microplastics (PS-MPs) promote NPC malignancy.

**Methods:**

Cellular uptake of PS-MPs was examined by confocal microscopy in NPC cells and NP69. Proliferation, migration, and invasion were evaluated by CCK-8, EdU, colony formation, wound healing, and Transwell assays. In vivo effects were tested in xenograft and lung-metastasis models with PS-MPs exposure via drinking water. Mechanistic investigations included RNA-seq, qRT-PCR, Western blot, ROS detection, immunofluorescence, and pharmacologic interventions with MitoTEMPO and ferroptosis inducers.

**Results:**

PS-MPs were readily internalized by NPC cells, with smaller particles showing higher uptake and NPC cells exhibiting greater uptake than NP69. Functionally, PS-MPs promoted proliferation, migration, invasion, and tumor progression. Mechanistically, they induced modest ROS accumulation, NRF2 nuclear translocation, and upregulation of SLC7A11 and GPX4, thereby suppressing ferroptosis. MitoTEMPO, but not DPI, reduced ROS and attenuated NRF2-SLC7A11/GPX4 signaling, indicating a mitochondrial origin of ROS. Importantly, ferroptosis restoration by Erastin or RSL3 reversed PS-MPs-induced malignant phenotypes and downregulated GPX4 and SLC7A11 expression.

**Conclusions:**

PS-MPs promote NPC progression by generating mitochondrial ROS that activate the NRF2-SLC7A11/GPX4 antioxidant axis and suppress ferroptosis. Pharmacologic reactivation of ferroptosis counteracts these effects, highlighting ferroptosis-targeted therapy as a potential strategy for mitigating microplastic-associated cancer risk.

**Supplementary Information:**

The online version contains supplementary material available at 10.1186/s12967-025-07508-w.

## Introduction

In recent years, MPs have emerged as a growing global environmental concern and have attracted increasing attention [1]. Defined as plastic particles smaller than 5 mm in diameter, MPs are widely distributed in water bodies, soil, air, and even living organisms [2]. With the surge in plastic consumption and the persistent nature of plastic degradation, MPs have become one of the major sources of environmental pollution. Their presence not only poses a significant threat to ecological systems but may also enter the human body through various routes, thereby posing potential health risks. Notably, in the field of cancer research, an increasing body of evidence suggests that MPs may affect the biological behavior of tumor cells through multiple mechanisms, contributing to tumor initiation and progression [3].

NPC is a malignant tumor of the head and neck region predominantly found in Southeast Asia and southern China [4]. Due to its insidious early symptoms, most patients are diagnosed at an advanced stage, often accompanied by high rates of metastasis and recurrence. The development of NPC is closely associated with genetic predisposition, Epstein-Barr virus (EBV) infection, and various environmental factors [5]. In recent years, growing attention has been paid to the potential impact of microplastics on NPC. Studies suggest that microplastics may accumulate in the body and affect cellular biological behavior by inducing inflammatory responses, oxidative stress, and inhibition of apoptosis, thereby creating favorable conditions for tumor initiation and progression [6]. Therefore, elucidating the role of microplastics in NPC—particularly their influence on intracellular redox balance, iron metabolism, and cell death pathways—is crucial for understanding their carcinogenic potential and their involvement in tumorigenesis.

Ferroptosis, a newly recognized form of regulated cell death, has garnered significant attention in various types of cancer [7]. Characterized by iron-dependent lipid peroxidation, ferroptosis plays a critical role in regulating tumor cell proliferation, survival, and metastasis [8, 9]. Studies have shown that inhibition of ferroptosis can promote tumor growth and metastatic potential, offering a novel therapeutic avenue for cancer treatment. Notably, the role of ROS in ferroptosis is dual-faceted-ROS can either trigger or suppress ferroptosis depending on their source, concentration, and the specific cellular context [10]. Microplastics may elevate intracellular ROS levels and activate NRF2-dependent antioxidant responses, including the upregulation of GPX4. When ROS levels remain within the cell’s compensatory threshold, such activation may paradoxically suppress ferroptosis [11]. However, this effect is likely influenced by factors such as the physicochemical properties of microplastics, exposure dose, and cell type. GPX4, a key antioxidant enzyme, protects cells from oxidative damage by reducing lipid peroxides [12]. The ROS-GPX4 axis has been widely implicated in ferroptosis regulation and plays a pivotal role in tumor cell proliferation and migration.

This study aims to investigate how microplastics promote the malignant progression of NPC by inhibiting ferroptosis. We hypothesize that microplastics may elevate intracellular ROS levels and disrupt iron metabolism pathways, thereby suppressing ferroptosis and conferring a growth advantage to NPC cells, ultimately facilitating their metastatic potential. This research not only provides new insights into the mechanisms underlying the health risks of microplastic exposure but also identifies potential therapeutic targets and novel strategies for the prevention and treatment of NPC.

## Materials and methods

### Cell lines

Human immortalized nasopharyngeal epithelial cell line NP69 and Nasopharyngeal carcinoma cell lines C666-1 and HONE1 were obtained from the Cell Center of Central South University. All NPC cell lines were cultured in RPMI-1640 (Life Technologies, Grand Island, NY, USA) in medium supplemented with 10% fetal bovine serum (FBS; Life Technologies) and 1% penicillin/streptomycin (Life Technologies). Cells were incubated at 37 °C in a humidified incubator with 5% CO_2_.

### Antibodies and reagents

Antibodies: Anti-GAPDH (#97166, CST), anti-GPX4 (Cat: ab125066, abcam), Anti-SLC7A11 (26864-1-AP, Proteintech), Anti-Nrf2(#12721, CST).

Reagents: Erastin (HY-15763, MCE), CCK-8 (HY-K0301, MCE), PS-MPs (SHANXI XINGBEI AIKE BIOTECHNOLOGY CO., LTD), Mito-TEMPO (HY-112879, MCE), DPI (HY-100965, MCE), U0126 (HY-12031 A, MCE) and EdU Staining Proliferation Kit (iFluor 647) (Cat: ab222421, abcam).

### Detection of microplastics into cells

The cells (NP69, C666-1 and HONE1) were seeded in culture dishes and allowed to adhere and grow until reaching approximately 70% confluency. The cells were then treated with serum-free medium containing red fluorescently labeled polystyrene microplastic particles (PS-MPs, 0.5–1 μm in diameter) at a concentration of 10 µg/mL for 24 h. After incubation, the cells were gently washed three times with pre-warmed PBS to remove non-internalized particles, followed by fixation with 4% paraformaldehyde for 10 min. Subsequently, the nuclei were stained with DAPI. After PBS washing, the cells were scanned and imaged using a laser confocal microscope.

### Detection of the IC₅₀ value of microplastics

The cells (C666-1 or HONE1) were seeded into 96-well plates at a density of approximately 5 × 10³ cells per well. After stable adherence, the cells were treated with serum-free medium containing different concentrations of red fluorescently labeled polystyrene microplastic particles (PS-MPs, 0.5 μm in diameter). The concentration range was set at 0, 50, 100, 150, 200, and 250 µg/mL, and cells were incubated for 24 h, with three replicates per group. After treatment, 10 µL of CCK-8 working solution was added to each well and incubated for an additional 1–2 h. The absorbance at 450 nm (OD₄₅₀) was measured using a microplate reader, and the relative cell viability was calculated using the untreated group as the control. A dose-response curve was plotted between microplastic concentration and cell viability, and the half maximal inhibitory concentration (IC₅₀) value was calculated by nonlinear regression fitting to evaluate the inhibitory effect of microplastics on cell proliferation.

### CCK-8

C666-1 or HONE1 cells were seeded into 96-well plates at a density of approximately 5 × 10³ cells per well. After stable adherence, the cells were treated with serum-free medium containing PS-MPs (0.5 μm in diameter) at their IC₅₀. Each group included three replicate wells, and the cells were incubated for 12 h, 24 h, 48 h, 60 h, and 72 h to assess time dependency. At the end of each time point, 10 µL of CCK-8 reagent was added to each well and incubated for an additional 1–2 h. The absorbance at 450 nm (OD₄₅₀) was then measured using a microplate reader.

### EdU assay

C666-1 and HONE1 nasopharyngeal carcinoma cells were seeded into culture plates and treated with PS-MPs after reaching the logarithmic growth phase, with untreated cells as the control group. EdU working solution (10 µM) was then added and incubated for 2–4 h to allow EdU incorporation into newly synthesized DNA of proliferating cells. After incubation, the cells were washed with PBS, fixed with 4% paraformaldehyde, and permeabilized with Triton X-100. A Click-iT reaction was performed according to the manufacturer’s protocol to conjugate fluorescent dyes to the incorporated EdU. Finally, nuclei were counterstained with DAPI, and images were acquired using a confocal fluorescence microscope. The percentage of EdU-positive cells was calculated to evaluate cell proliferation.

### Wound healing assay

C666-1 or HONE1 cells were seeded into 6-well plates and cultured until reaching approximately 90% confluency. A straight wound was created vertically across the center of each well using a sterile pipette tip. Detached cells were removed, and the wells were gently washed 2–3 times with PBS. The cells were then treated with serum-free medium containing PS-MPs (0.5 μm in diameter) at their IC₅₀, with an untreated group set as control. Images of the scratch area were captured at 0 h and 48 h to assess wound closure. ImageJ software was used to measure the scratch width, and the relative migration rate was calculated.

### Migration assay

Cells pretreated for 24 h (C666-1 or HONE1) were resuspended in serum-free medium and adjusted to a concentration of 2 × 10⁵ cells/mL. A total of 200 µL of the serum-free cell suspension containing PS-MPs (0.5 μm in diameter) at the IC₅₀ was added to the upper chamber of the Transwell insert, while 600 µL of complete medium containing 10% fetal bovine serum was added to the lower chamber as a chemoattractant. An untreated control group was included. The Transwell plate was incubated at 37 °C with 5% CO₂ for 48 h. After incubation, non-migrated cells on the upper surface of the membrane were gently removed with a cotton swab and washed twice with PBS. Cells that had migrated to the lower surface were fixed with 4% paraformaldehyde for 10 min and stained with 0.1% crystal violet for 20 min. After washing with PBS, five random fields were selected under a microscope to count the number of migrated cells, to evaluate the effect of microplastics on cell migration.

### Invasion assay

The upper surface of the Transwell insert membrane was evenly coated with Matrigel (diluted, approximately 50 µL per well) and incubated at 37 °C for 1 h to allow gel solidification. Cells pretreated for 24 h (C666-1 or HONE1) were resuspended in serum-free medium and adjusted to a concentration of 2 × 10⁵ cells/mL. A total of 200 µL of the cell suspension containing PS-MPs (0.5 μm in diameter) at the IC₅₀ was added to the upper chamber, while 600 µL of complete medium containing 10% fetal bovine serum was added to the lower chamber as a chemoattractant. An untreated control group was included. The Transwell plate was incubated at 37 °C with 5% CO₂ for 48 h. After incubation, non-invaded cells on the upper surface of the membrane were gently removed with a cotton swab and washed twice with PBS. Cells that had invaded to the lower surface of the membrane were fixed with 4% paraformaldehyde for 10 min and stained with 0.1% crystal violet for 20 min. After washing with PBS, five random fields were selected under a microscope to count the number of invaded cells, to evaluate the effect of microplastics on cell invasion.

### Clone formation assay

The cells (C666-1 or HONE1) were seeded at a low density (approximately 500–1000 cells per well) into 6-well plates. After the cells adhered, they were treated with serum-free medium containing PS-MPs (0.5 μm in diameter) at the (IC₅₀), with an untreated control group included. The cells were continuously cultured at 37 °C in a 5% CO₂ incubator for 10–14 days, with the medium containing microplastics refreshed every 2–3 days. Once visible colonies had formed, the medium was discarded, and the wells were gently washed with PBS, followed by fixation with 4% paraformaldehyde for 15 min and staining with 0.1% crystal violet for 20 min. After staining, the wells were washed thoroughly, photographed, and the number of colonies was quantified using ImageJ.

### Xenograft model

To evaluate the effect of microplastics on the in vivo proliferative capacity of nasopharyngeal carcinoma cells, a subcutaneous xenograft model in nude mice was established. C666-1 cells were collected and resuspended in PBS at a concentration of 5 × 10⁷ cells/mL. Each BALB/c nude mouse was subcutaneously injected with 100 µL of the cell suspension (a total of 5 × 10⁶ cells) into the dorsal region. PS-MPs (0.5 μm in diameter) were administered via drinking water at a daily intake of approximately 250 µg per mice, and the mice were continuously treated for about 5 weeks. At the end of the experiment, the animals were sacrificed, and the tumors were excised, weighed, and photographed for analysis.

To evaluate the effect of microplastics on the in vivo metastatic potential of nasopharyngeal carcinoma cells, a tail vein injection lung metastasis model in nude mice was established. C666-1 cells were pretreated for 24 h, then collected and resuspended in PBS at a concentration of 2 × 10⁶ cells/mL. Four-week-old female BALB/c nude mice were injected via the tail vein with 100 µL of the cell suspension (a total of 2 × 10⁵ cells). After injection, the mice were maintained for 5 weeks, during which PS-MPs (0.5 μm in diameter) were administered through drinking water at a daily intake of approximately 250 µg per mouse. The general condition and body weight of the mice were monitored throughout the experiment. At the end of the study, the animals were sacrificed, and lung tissues were harvested, photographed, and fixed in 4% paraformaldehyde. The tissues were then embedded in paraffin and subjected to hematoxylin and eosin (HE) staining. The number and area of metastatic nodules in the lungs were observed and quantified to assess the impact of microplastics on tumor cell lung metastasis.

To evaluate the combined effect of microplastics and Erastin on the in vivo proliferative capacity of nasopharyngeal carcinoma cells, a subcutaneous xenograft model was established using BALB/c nude mice. C666-1 cells were collected and suspended in phosphate-buffered saline (PBS) at a concentration of 5 × 10⁷ cells/mL. Each mouse was subcutaneously injected with 100 µL of the cell suspension into the dorsal region, delivering a total of 5 × 10⁶ cells per mouse. PS-MPs (0.5 μm in diameter) were administered via drinking water at a dose of approximately 250 µg per mouse per day for a duration of 5 weeks. Once the tumor volume reached approximately 80 mm³, intervention was initiated. In the PS-MPs + Erastin group, mice continued to receive PS-MPs and were additionally administered Erastin via intraperitoneal injection at 10 mg/kg daily [13]. Mice in the Control and PS-MPs groups received intraperitoneal injections of an equal volume of PBS as a control. The intervention lasted for 3 weeks, after which the animals were sacrificed. Tumors were excised, weighed, photographed, and subjected to further analyses.

### Immunohistochemistry

To assess the expression level of Ki67 in tumor tissues from nude mice, immunohistochemical (IHC) staining was performed. Tumor samples were fixed in 4% paraformaldehyde for 24 h, followed by routine dehydration, paraffin embedding, and sectioning. The tissue sections were baked at 65 °C, deparaffinized with xylene, and rehydrated through a graded ethanol series. Antigen retrieval was carried out using citrate buffer at 95–100 °C for 15–20 min. After cooling, endogenous peroxidase activity was blocked with 3% hydrogen peroxide for 10 min at room temperature, followed by blocking of nonspecific binding with 5% BSA. The sections were then incubated with an anti-Ki67 primary antibody overnight at 4 °C. After washing with PBS the next day, HRP-conjugated secondary antibody was added and incubated at 37 °C for 30 min. Visualization was performed using DAB chromogen, followed by hematoxylin counterstaining, dehydration, clearing, and mounting. Ki67-positive cells were observed under a microscope, and the positive rate was quantified using ImageJ software to evaluate cell proliferation activity.

### Hematoxylin and eosin (HE) staining

To evaluate the formation of metastatic tumor nodules in the lung tissue of nude mice, HE staining was performed for histological analysis. At the end of the experiment, the mice were sacrificed, and lung tissues were immediately harvested and fixed in 4% paraformaldehyde for 24 h. The fixed lungs were then subjected to routine dehydration, paraffin embedding, and sectioning. The tissue sections were baked at 65 °C, deparaffinized with xylene, and rehydrated through a graded ethanol series. Nuclear staining was performed with hematoxylin for 5–10 min, followed by water washing, differentiation with acid alcohol, bluing in alkaline water, and eosin counterstaining. The sections were then dehydrated, cleared, and mounted. Under a microscope, the lung architecture and distribution of tumor nodules were examined. Metastatic nodules appeared as irregularly shaped, densely packed tumor cell clusters with well-defined boundaries located within normal alveolar tissue. Multiple random fields were selected to count metastatic foci, to assess the effect of microplastic exposure on tumor lung metastasis.

### Western blotting

Equal amounts of cell lysates were loaded into each well of the SDS-PAGE gel for electrophoresis. Following separation, the proteins were transferred onto PVDF membranes and blocked with non-fat milk to prevent nonspecific binding. The membranes were subsequently incubated with the specified primary antibodies, followed by horseradish peroxidase (HRP)-conjugated secondary antibodies. Protein signals were visualized using an enhanced chemiluminescence (ECL) detection system.

### RNA extraction and qRT-PCR

Total RNA was extracted using TRIzol reagent (Invitrogen, CA, USA) following the manufacturer’s protocol. One microgram of total RNA was reverse transcribed into complementary DNA (cDNA) using the HiScript II Q RT SuperMix for qPCR (Vazyme, Nanjing, China). Quantitative PCR was then performed using 2× SYBR Green qPCR Master Mix (Bimake, Changsha, China). All primers were synthesized by Tsingke (Changsha, China), and their sequences are listed in table S1.

### Detection of ROS

The cells (C666-1 or HONE1) treated with PS-MPs were gently washed twice with PBS, then incubated with serum-free medium containing 10 µM DCFH-DA (Beyotime, China) at 37 °C for 30 min, with gentle shaking to facilitate uniform probe uptake. After incubation, the cells were washed three more times with PBS to remove excess probe and minimize background fluorescence. The intracellular ROS levels were then observed using a laser confocal microscope.

Subcutaneous tumor tissues from nude mice were immediately processed into frozen sections (5–8 μm thick), placed on glass slides, and air-dried at room temperature for fixation. The sections were washed 2–3 times with PBS to remove residual OCT, then incubated with 10 µM DCFH-DA in serum-free medium at 37 °C for 20–30 min in the dark, allowing the probe to penetrate cells and be oxidized into a fluorescent product. After incubation, sections were washed 2–3 times with PBS to remove excess probe and minimize background. Green fluorescence was then detected using a confocal laser scanning microscope with 488 nm excitation, and fluorescence intensity was compared between groups to assess ROS levels.

### RNA sequencing

Total RNA was extracted from C666-1 cells treated with PS-MPs (IC₅₀, 24 h) and untreated control cells using TRIzol reagent (Invitrogen, USA), according to the manufacturer’s instructions. Transcriptome sequencing was performed on the Illumina PE150 platform (HaploX, China). Differentially expressed genes were defined as those with a |log₂ fold change| ≥ 2 and a *p*-value < 0.05.

### Statistical analysis

All data were statistically analyzed using GraphPad Prism 8 software. Experimental data are expressed as the mean ± S.E.M. Statistical significance of the data was analyzed by standard Student’s t test. A *p* < 0.05 was considered statistically significant, and *p* < 0.01 was considered statistically significant.

## Results

### Differences in cellular uptake of PS-MPs with different particle sizes

**Fig. 1:**
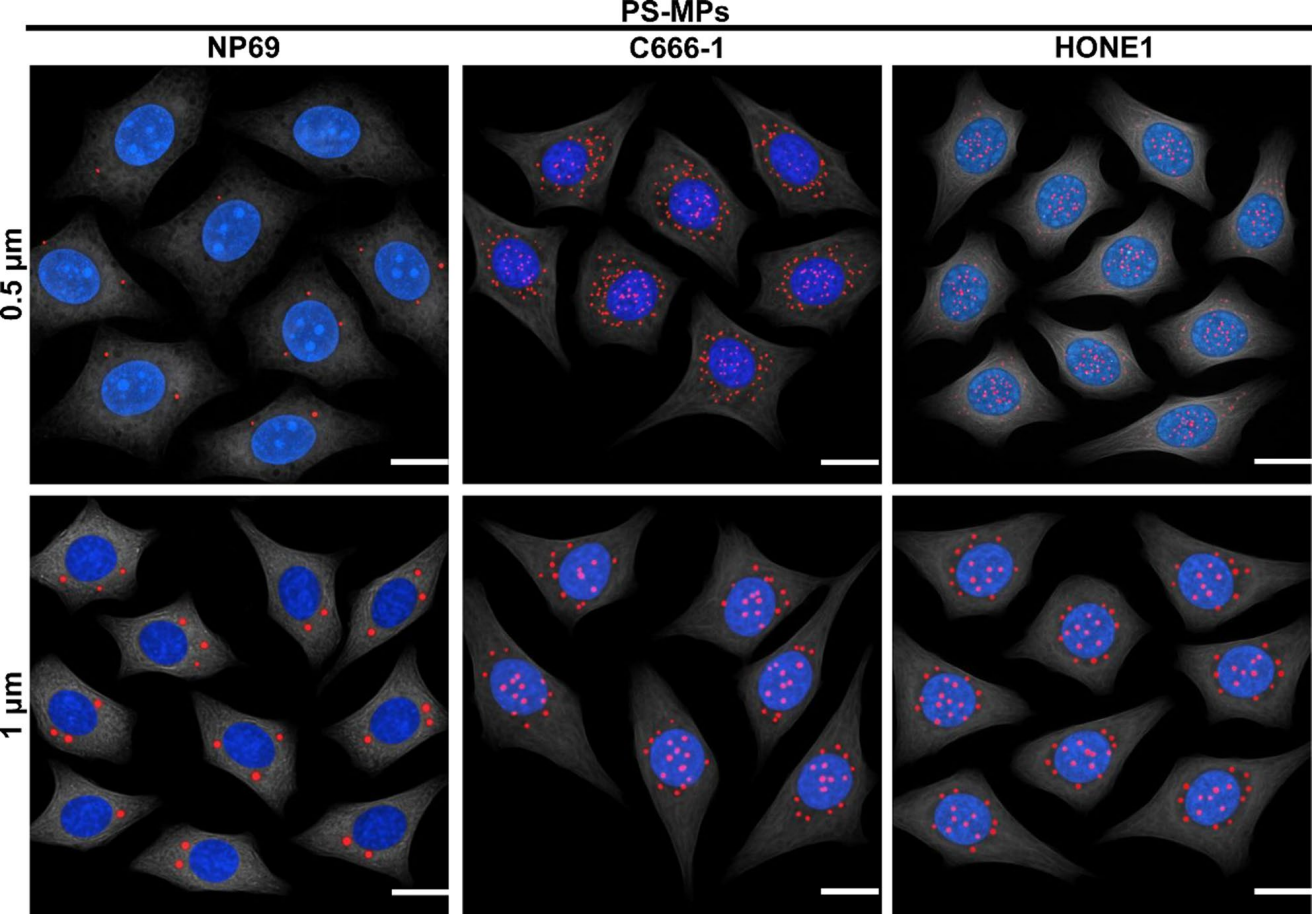
Size- and cell type-dependent uptake of PS-MPs in cells. Confocal microscopy images show the internalization of red fluorescently labeled PS-MPs (0.5–1 μm, 10 µg/mL, 24 h) in C666-1, HONE1, and NP69 cells. Nasopharyngeal carcinoma cells (C666-1 and HONE1) exhibited markedly higher uptake efficiency than NP69, with smaller 0.5 μm PS-MPs internalized more efficiently than 1 μm particles. Red fluorescence represents PS-MPs, while blue fluorescence (DAPI) marks cell nuclei

To evaluate the internalization efficiency of PS-MPs of different sizes in nasopharyngeal carcinoma cells, C666-1 or HONE1 cells were incubated with red fluorescently labeled PS-MPs of 0.5 μm and 1 μm diameters at a concentration of 10 µg/mL for 24 h. As shown in the confocal fluorescence images (Fig. 1), PS-MPs (red) were clearly distributed in the cytoplasmic region surrounding the DAPI-stained nuclei (blue), indicating successful cellular uptake. Notably, cells treated with 0.5 μm PS-MPs exhibited a higher intracellular fluorescence intensity and a greater number of internalized particles compared to those treated with 1 μm PS-MPs, suggesting that smaller PS-MPs are taken up more efficiently by nasopharyngeal carcinoma cells under the same exposure conditions.

In addition, a comparative analysis with the immortalized normal nasopharyngeal epithelial cell line NP69 revealed a distinct difference in uptake capacity. Both C666-1 and HONE1 cells showed markedly higher levels of PS-MPs internalization than NP69, regardless of particle size (Fig. 1). This indicates that nasopharyngeal carcinoma cells possess an enhanced ability to internalize microplastic particles, which may reflect alterations in their endocytic activity or cytoskeletal dynamics associated with malignant transformation. Together, these findings demonstrate that the efficiency of PS-MPs uptake is influenced not only by particle size but also by the intrinsic properties of tumor versus normal cells.

### PS-MPs promote cell proliferation and colony formation in NPC cells

**Fig. 2:**
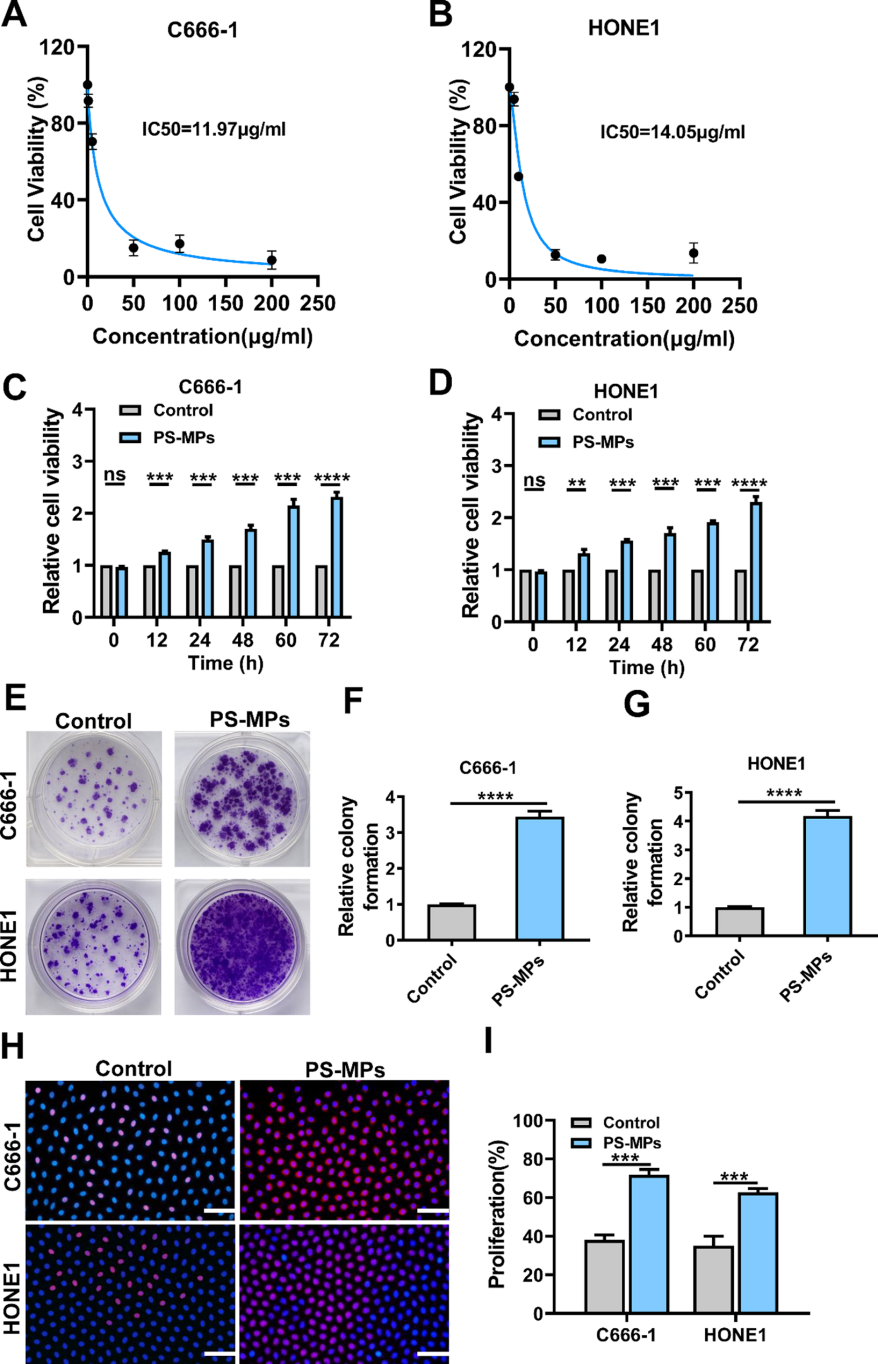
PS-MPs enhance proliferation and colony-forming ability of NPC cells. (**A**-**B**) CCK-8 assay showing dose-dependent increase in cell viability following treatment with various concentrations of PS-MPs, with the IC₅₀ value calculated accordingly. (**C**-**D**) Time-course CCK-8 analysis demonstrating that PS-MPs at IC₅₀ concentration promote cell proliferation over 12 to 72 h. (**E**-**G**) Colony formation assay indicating that PS-MP treatment significantly increases the number and size of cell colonies compared to the control group. (**H**-**I**) EdU incorporation assay showing a higher proportion of EdU-positive nuclei (red) in PS-MP-treated NPC cells compared with the control group, confirming enhanced DNA replication and proliferative activity. Red fluorescence represents EdU-positive nuclei, while blue fluorescence (DAPI) marks total nuclei. Each experiment was performed in triplicate (*n* = 3). *p < 0.05, **p < 0.01, ***p < 0.001, ****p < 0.0001

To evaluate the biological effects of PS-MPs on NPC cells, a series of proliferation assays were conducted. As shown in Fig. 2A-B, cell viability increased with rising concentrations of PS-MPs (0–250 µg/mL), and the IC₅₀ was accordingly determined. Based on this IC₅₀ value, a time-course study was performed to further investigate the time-dependent proliferative effect of PS-MPs (Fig. 2C-D). The results showed a gradual increase in cell viability from 12 to 72 h, indicating that the proliferative effect of PS-MP exposure is positively correlated with exposure duration.

To assess the long-term proliferative potential, a colony formation assay was carried out under IC₅₀ conditions. As shown in Fig. 2E-G, cells treated with PS-MPs formed significantly more and larger colonies compared to the untreated group, suggesting that PS-MPs not only enhance short-term cell viability but also promote the clonogenic capacity of NPC cells.

Furthermore, an EdU incorporation assay was performed to directly evaluate DNA synthesis and cell proliferation activity (Fig. 2H-I). Consistent with the CCK-8 and colony formation results, PS-MP exposure led to a significantly higher proportion of EdU-positive nuclei (red), indicating enhanced DNA replication and active proliferation in NPC cells. Together, these findings demonstrate that PS-MPs effectively promote both short-term proliferation and long-term colony formation in NPC cells, highlighting their tumor-promoting potential.

### PS-MPs enhance invasion and migration of NPC cells

**Fig. 3:**
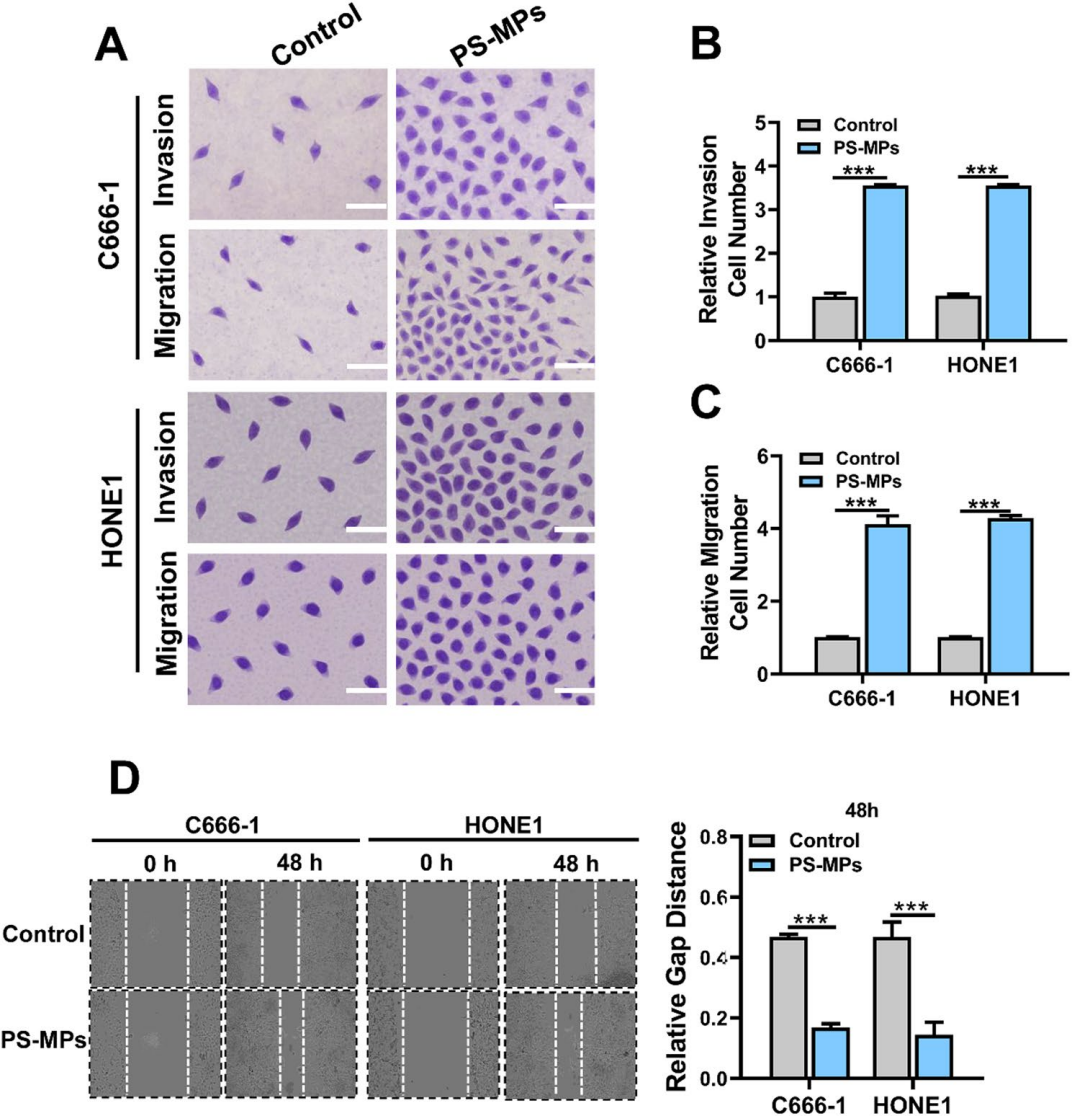
PS-MPs promote migration and invasion of NPC cells. (**A**–**C**) Transwell invasion and migration assays showing that PS-MP-treated NPC cells exhibit significantly increased invasive and migratory abilities compared to the control group. (**D**) Wound healing assay demonstrating enhanced scratch closure in PS-MP-treated cells, indicating increased lateral migration capacity. Each experiment was performed in triplicate (*n* = 3). *p < 0.05, **p < 0.01, ***p < 0.001, ****p < 0.0001

To investigate the effects of PS-MPs on the invasiveness and migratory capacity of nasopharyngeal carcinoma cells, Transwell assays were performed. As shown in Fig. 3A-C, both migration and invasion assays demonstrated a significant increase in the number of migrated and invaded cells in the PS-MP-treated group compared to the untreated control group, indicating that PS-MPs promote cellular migration and invasion.

In addition, a wound healing assay was conducted to assess the lateral migratory ability of the cells (Fig. 3D). PS-MP-treated cells exhibited accelerated scratch closure over time, further confirming that PS-MPs enhance the migratory capacity of nasopharyngeal carcinoma cells.

### PS-MPs promote in vivo growth and metastasis of NPC cells

**Fig. 4:**
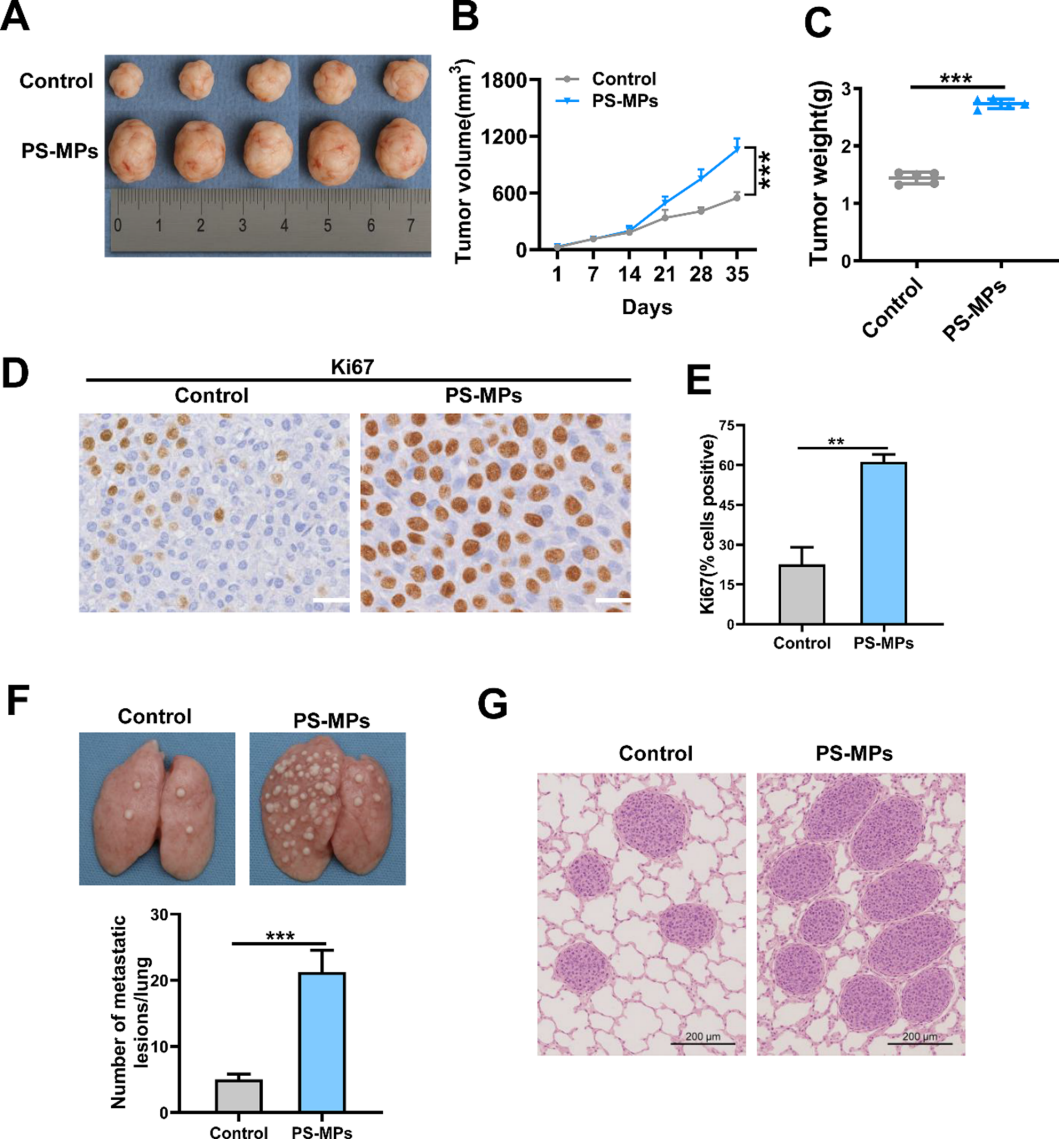
PS-MPs promote in vivo tumor growth and lung metastasis of NPC cells. (**A**–**C**) Subcutaneous xenograft model in BALB/c nude mice injected with C666-1 cells. Mice were given drinking water with or without polystyrene microplastics (PS-MPs, 0.5 μm, ~ 250 µg/day) for 5 weeks. Representative images of excised tumors (**A**), tumor volume growth curves (**B**), and final tumor weights (**C**) are shown. (**D**–**E**) Immunohistochemical staining for Ki67 in tumor tissues. Representative images (**D**) and quantification of Ki67-positive cells (**E**) demonstrate increased proliferation in the PS-MP-treated group. (**F**–**G**) Lung metastasis model via tail vein injection of C666-1 cells. Mice were exposed to PS-MPs via drinking water (~ 250 µg/day) for 5 weeks. Representative lung photographs and quantification of metastatic nodules show enhanced pulmonary metastasis in the PS-MP group. *n* = 5 mice per group. *p < 0.05, **p < 0.01, ***p < 0.001, ****p < 0.0001

To evaluate the in vivo effects of PS-MPs on the progression of nasopharyngeal carcinoma, both subcutaneous xenograft and tail vein lung metastasis models were established in BALB/c nude mice. As shown in Fig. 4A-C, mice that received drinking water supplemented with PS-MPs exhibited significantly increased tumor volume and weight compared to the control group, indicating that PS-MPs promote tumor growth in vivo. To further confirm their effect on tumor cell proliferation, immunohistochemical staining for the proliferation marker Ki67 was performed (Fig. 4D-E). Tumor tissues from the PS-MP-treated group showed a marked increase in Ki67-positive cells, suggesting enhanced proliferative activity.

In addition, tail vein injection experiments were conducted to assess the metastatic potential (Fig. 4F-G). Compared to the control group, mice exposed to PS-MPs via drinking water developed a greater number of visible metastatic nodules in the lungs. Histological analysis confirmed increased pulmonary metastasis in the PS-MP-treated group, indicating that microplastic exposure facilitates the lung colonization capacity of nasopharyngeal carcinoma cells.

### PS-MPs May suppress ferroptosis-related pathways via ROS modulation

**Fig. 5:**
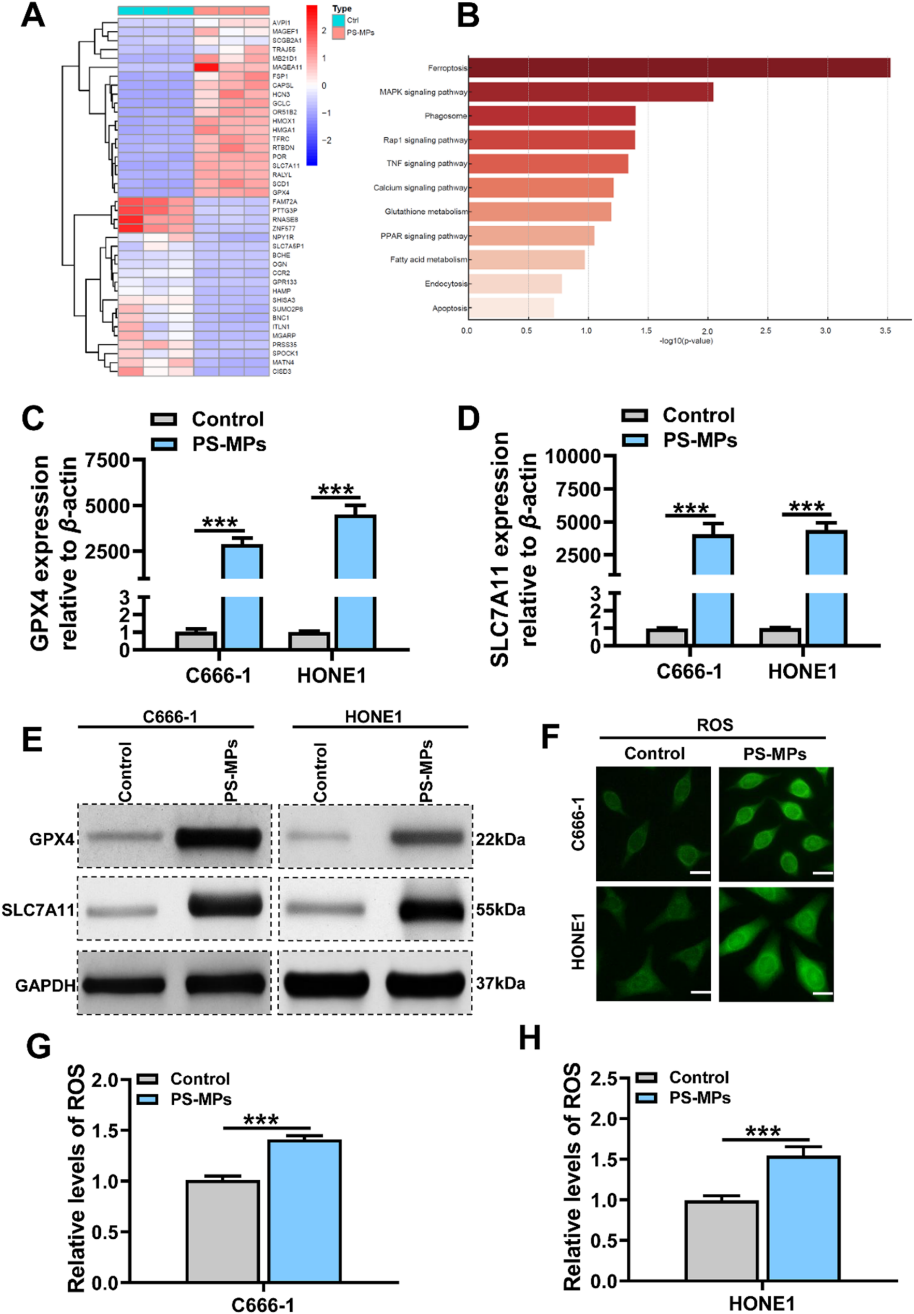
PS-MPs activate ferroptosis-related pathways and moderately elevate ROS levels in NPC cells. (**A**) Heatmap of differentially expressed genes between PS-MP-treated and control C666-1 cells, showing distinct transcriptomic profiles. (**B**) KEGG pathway enrichment analysis indicating significant involvement of ferroptosis-related pathways in the PS-MPs-treated group. (**C**–**D**) qRT-PCR analysis of mRNA expression levels of key ferroptosis-related genes, showing upregulation upon PS-MP treatment. (**E**) Western blot analysis confirming increased protein expression of ferroptosis markers in PS-MP-treated cells. (**F**–**H**) Intracellular ROS levels were measured using the DCFH-DA fluorescent probe. PS-MPs exposure resulted in a slight increase in green fluorescence intensity, suggesting a moderate elevation in ROS production. Each experiment was performed in triplicate (*n* = 3). *p < 0.05, **p < 0.01, ***p < 0.001, ****p < 0.0001

To explore the potential mechanisms by which PS-MPs affect nasopharyngeal carcinoma cells, transcriptomic and functional analyses were conducted. As shown in Fig. 5A, hierarchical clustering heatmaps revealed distinct gene expression profiles between PS-MP-treated C666-1 cells and controls. Pathway enrichment analysis (Fig. 5B) indicated significant enrichment of ferroptosis-related signaling pathways, suggesting that PS-MPs may be involved in the regulation of ferroptosis responses.

To assess the extent of ferroptosis involvement, the mRNA expression levels of key ferroptosis-related genes were quantified by qRT-PCR (Fig. 5C-D). The results showed a marked upregulation of these genes upon PS-MP exposure. Consistently, Western blot analysis (Fig. 5E) confirmed increased protein levels of ferroptosis markers in the PS-MP-treated group compared to controls.

Intracellular reactive oxygen species (ROS) levels were further evaluated using the DCFH-DA fluorescent probe (Fig. 5F-H). A slight increase in ROS fluorescence was observed in cells exposed to PS-MPs compared to untreated controls. Although the elevation was modest, it suggests that PS-MP-induced suppression of ferroptosis may be associated with disturbed ROS homeostasis, implying a potential ROS-mediated regulatory mechanism.

To further investigate whether MAPK signaling contributes to PS-MPs-induced NRF2 activation, NPC cells were treated with the MAPK pathway inhibitor U0126. As shown in Figure S1, inhibition of MAPK signaling did not reverse the nuclear translocation of NRF2 triggered by PS-MPs. These findings suggest that PS-MPs promote NRF2 activation through a MAPK-independent mechanism.

### Mitochondria-derived ROS mediate PS-MP-induced NRF2 activation and upregulation of SLC7A11/GPX4

**Fig. 6:**
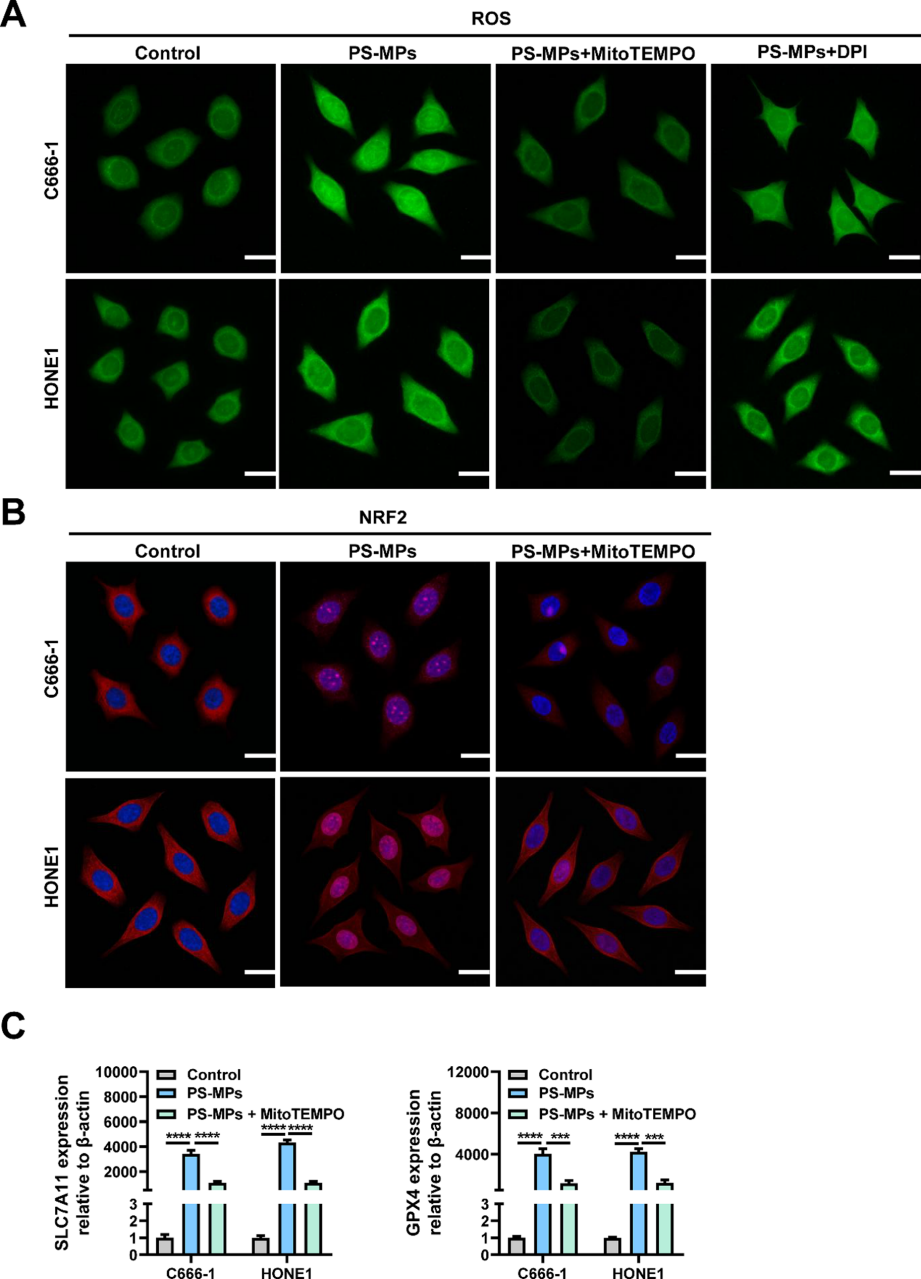
Mitochondria-derived ROS drive NRF2 activation and SLC7A11/GPX4 expression in NPC cells. (**A**) DCFH-DA assay showing mitochondrial ROS as the major source of PS-MP-induced ROS. (**B**) Immunofluorescence of NRF2 nuclear translocation with or without MitoTEMPO. (**C**) qRT-PCR of SLC7A11 and GPX4 expression. Each experiment was performed in triplicate (*n* = 3). *p < 0.05, **p < 0.01, ***p < 0.001, ****p < 0.0001

To identify the source of ROS following PS-MP exposure, C666-1 and HONE1 cells were treated with PS-MPs in the presence of either MitoTEMPO (a mitochondrial-targeted ROS scavenger) or DPI (an inhibitor of NADPH oxidase). As shown in Fig. 6A, ROS levels were markedly reduced only in the PS-MPs + MitoTEMPO group, while no significant reduction was observed with DPI treatment. These results indicate that mitochondria are the predominant source of ROS induced by PS-MPs.

Furthermore, PS-MPs exposure promoted nuclear translocation of NRF2 (Fig. 6B), accompanied by increased transcriptional expression of its downstream antioxidant genes, SLC7A11 and GPX4 (Fig. 6C). Importantly, co-treatment with MitoTEMPO suppressed both NRF2 nuclear accumulation and the transcriptional upregulation of SLC7A11 and GPX4(Fig. 6B-C), suggesting that mitochondria-derived ROS play a pivotal role in activating the NRF2-SLC7A11/GPX4 axis. Collectively, these data demonstrate that PS-MPs stimulate mitochondrial ROS production, which in turn drives NRF2-mediated antioxidant responses in NPC cells.

### Ferroptosis inducers reverse PS-MPs-induced proliferative and metastatic phenotypes in NPC cells

**Fig. 7:**
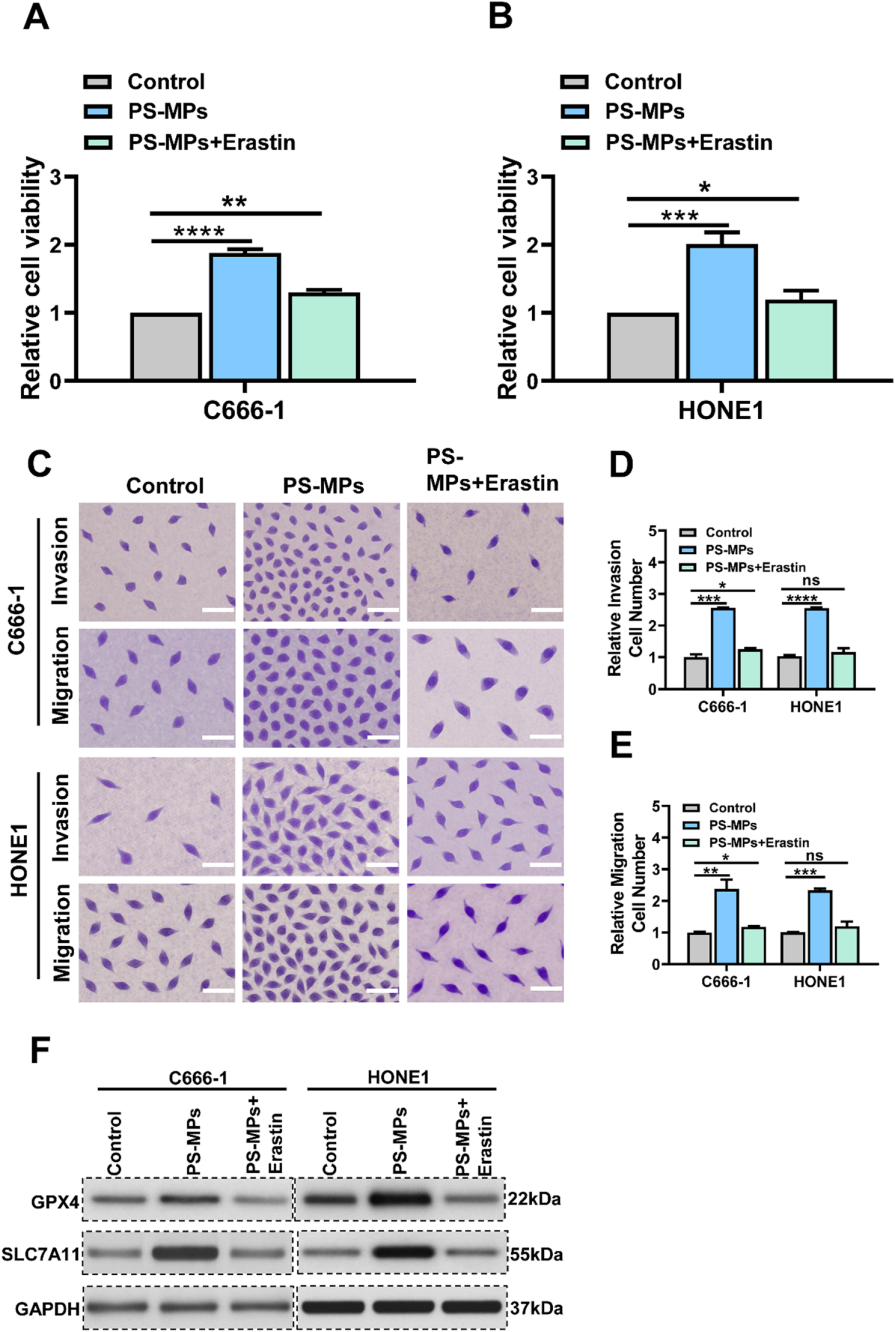
Erastin reverses the tumor-promoting effects of PS-MPs by reactivating ferroptosis. (**A**–**B**) CCK-8 assay showing that Erastin treatment attenuates the PS-MP-induced increase in cell proliferation. (**C**-**E**) Transwell migration and invasion assays demonstrating that Erastin significantly reduces the enhanced migratory and invasive abilities induced by PS-MP exposure. (**F**) Western blot analysis showing that Erastin reverses the upregulation of ferroptosis-related proteins induced by PS-MPs. Each experiment was performed in triplicate (*n* = 3). *p < 0.05, **p < 0.01, ***p < 0.001, ****p < 0.0001

To investigate whether ferroptosis is mechanistically involved in PS-MP-induced tumor-promoting effects, we first performed rescue experiments using the ferroptosis inducer Erastin. As shown in Fig. 7A-B, CCK-8 assays demonstrated that Erastin significantly suppressed the enhanced proliferation of nasopharyngeal carcinoma cells induced by PS-MPs. Similarly, Transwell assays revealed that Erastin markedly reduced the migratory and invasive capacities of PS-MP-treated cells (Fig. 7C-E). At the molecular level, Western blot analysis further showed that Erastin treatment decreased the expression of ferroptosis suppressors GPX4 and SLC7A11, which had been upregulated by PS-MPs (Fig. 7F). These findings indicate that Erastin restores ferroptosis activity and counteracts PS-MP-mediated malignant phenotypes.

To further validate the role of ferroptosis, cells were treated with the GPX4 inhibitor RSL3. Consistent with the results obtained using Erastin, RSL3 treatment effectively reversed PS-MPs-induced proliferation, migration, and invasion (Fig. S2A-C). Collectively, these results provide strong evidence that ferroptosis suppression is a key mechanism by which PS-MPs promote tumor progression, and that restoring ferroptosis sensitivity—either by Erastin or RSL3—can effectively reverse the malignant phenotypes induced by PS-MPs in NPC cells.

### Erastin reverses PS-MPs-mediated ferroptosis Inhibition and suppresses in vivo proliferation of NPC cells

**Fig. 8:**
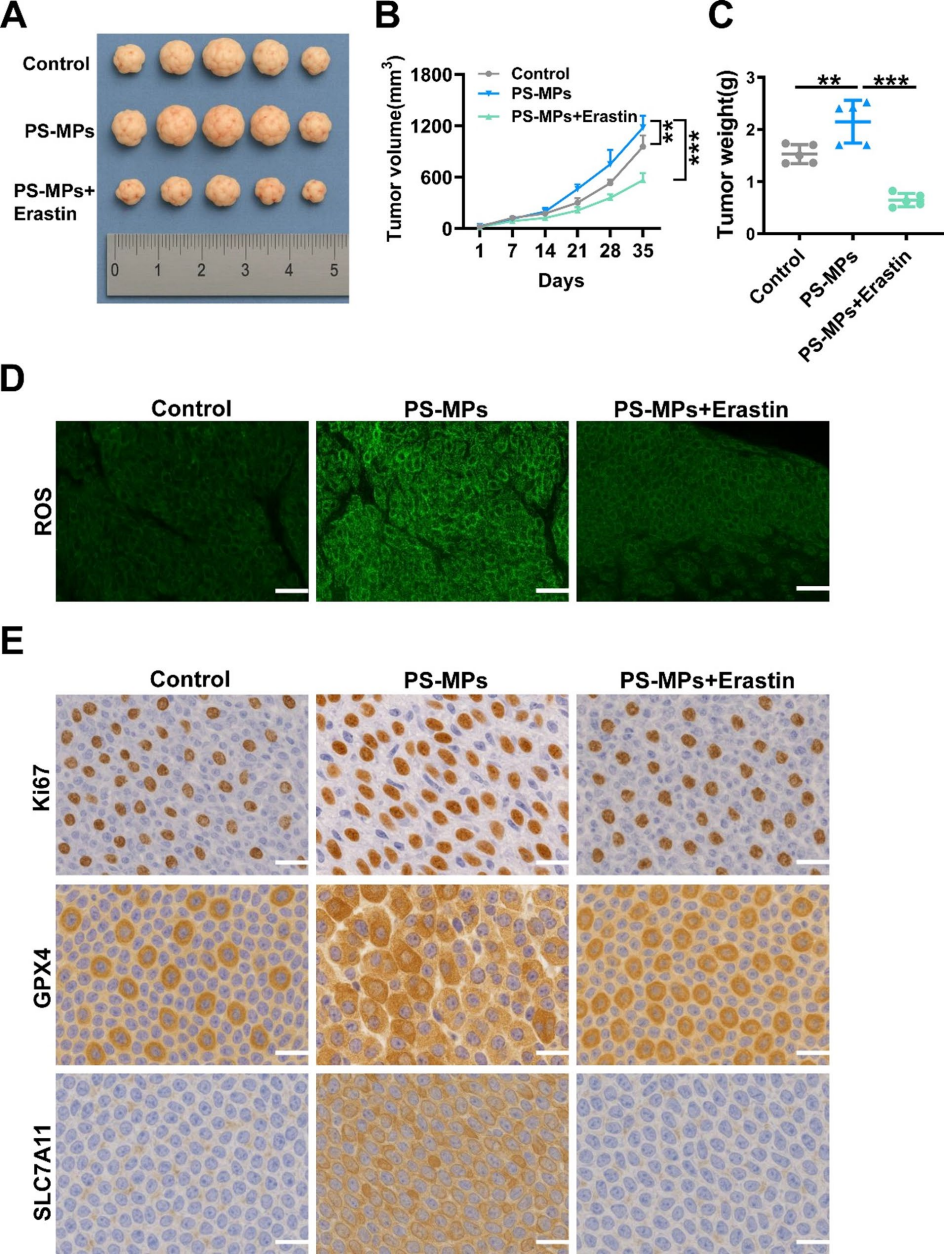
Effects of PS-MPs and Erastin on tumor growth and marker expression in vivo. (**A**) Representative images of subcutaneous xenograft tumors derived from C666-1 cells in BALB/c nude mice after 5 weeks of treatment. (**B**) Tumor volume was measured and presented as growth curves. (**C**) Final tumor weights were measured after euthanasia. (**D**) DCFH-DA staining of frozen tumor sections showing ROS levels in different groups. (**E**) Immunohistochemical staining of Ki67, GPX4, and SLC7A11 in tumor tissues from each treatment group. Ki67 staining indicates proliferative activity; GPX4 and SLC7A11 reflect ferroptosis-related molecular changes. *n* = 5 mice per group. *p < 0.05, **p < 0.01, ***p < 0.001, ****p < 0.0001

To investigate the combined effects of PS-MPs and the ferroptosis inducer Erastin on tumor growth in vivo, a subcutaneous xenograft model was established using C666-1 nasopharyngeal carcinoma cells in BALB/c nude mice. As shown in Fig. 8A, tumor size in the PS-MPs group was markedly larger than that in the control group, while co-treatment with Erastin significantly reduced tumor volume. Tumor growth curves revealed that PS-MPs notably promoted tumor progression, whereas Erastin intervention effectively suppressed this effect (Fig. 8B). Final tumor weights exhibited a consistent trend, with the PS-MPs + Erastin group showing significantly lower tumor weights compared to the PS-MPs group (Fig. 8C).

ROS levels in tumor tissues were further examined using DCFH-DA fluorescence staining (Fig. 8D). Compared with the control group, the PS-MPs group displayed significantly elevated ROS accumulation, while co-treatment with Erastin partially reduced ROS levels, suggesting that Erastin restored redox balance by counteracting PS-MP-induced oxidative stress.

Immunohistochemical analysis demonstrated that the expression of the proliferation marker Ki67 was elevated in the PS-MPs group, indicating enhanced tumor cell proliferation, but was significantly downregulated upon Erastin treatment (Fig. 8E). Regarding ferroptosis-related markers, PS-MPs exposure upregulated the expression of GPX4 and SLC7A11, suggesting inhibition of the ferroptosis pathway and enhanced tumor cell survival. In contrast, combined treatment with Erastin markedly reduced GPX4 and SLC7A11 levels, indicating that Erastin could reverse PS-MPs-induced ferroptosis suppression and exert anti-tumor effects (Fig. 8E).

In summary, PS-MPs promote tumor growth in nasopharyngeal carcinoma by inhibiting ferroptosis and inducing aberrant ROS accumulation, while Erastin effectively counteracts these effects by restoring ferroptosis sensitivity, reducing ROS, and suppressing tumor progression.

## Discussion

This study systematically investigated the uptake characteristics of PS-MPs in NPC cells and their regulatory effects on cell proliferation, migration, invasion, and ferroptosis-related pathways. The findings reveal a potential mechanism by which microplastics promote the malignant progression of NPC by suppressing ferroptosis. This research provides experimental evidence supporting a causal link between microplastic exposure and tumor development, suggesting that microplastics are not only ecological risk factors but may also serve as environmental drivers of cancer progression.

Firstly, confocal laser scanning microscopy clearly demonstrated that PS-MPs are efficiently internalized by nasopharyngeal carcinoma cells, with smaller particles showing more pronounced intracellular accumulation. This observation aligns with previous studies on nanoparticle uptake pathways, such as pinocytosis, macropinocytosis, and active transport, all of which are highly sensitive to particle size [14–16]. The higher internalization efficiency of smaller particles may accelerate the release of oxidative stress within cells, disrupt membrane systems and signaling pathways, and thereby enhance their biological activity.

In terms of functional phenotypes, PS-MPs significantly promoted the proliferation, colony-forming ability, migration, and invasion of nasopharyngeal carcinoma cells. This tumor-promoting effect was further validated in animal models, where PS-MP exposure led to increased subcutaneous tumor volume, elevated Ki67 expression, and a marked rise in pulmonary metastatic nodules in nude mice. Notably, these results were obtained in the absence of other carcinogenic stimuli, suggesting that PS-MPs may possess intrinsic “pro-tumor” or “tumor-promoting” properties. Considering the reality of environmental exposure, this finding holds important public health implications.

Mechanistic studies revealed that PS-MPs can activate a range of signaling pathways associated with the suppression of ferroptosis. Ferroptosis is a distinct form of programmed cell death, characterized by the iron-dependent accumulation of lipid peroxides. GPX4 and SLC7A11 are key regulators that maintain cellular resistance to ferroptosis by eliminating phospholipid hydroperoxides and sustaining glutathione synthesis, respectively [17–19]. In this study, both proteins were significantly upregulated following PS-MP exposure, suggesting that the antioxidant defense system was activated to counteract the potential threat of ferroptosis.

Notably, although reactive oxygen species (ROS)—a key upstream trigger of ferroptosis—were slightly elevated following PS-MP exposure, the increase did not reach the threshold required to induce cell death. Instead, it may have activated antioxidant factors such as NRF2, leading to the upregulation of GPX4 and initiating a “compensatory suppression of ferroptosis” [20]. This regulatory axis involving ROS, antioxidant response, and ferroptosis has been reported in various stress contexts [21–24], suggesting that ferroptosis can be actively suppressed as part of a cellular defense mechanism, yet may be rapidly executed once the antioxidant barrier is breached.

To verify the functional role of ferroptosis in the tumor-promoting effects of PS-MPs, we employed Erastin, a classical ferroptosis inducer, for intervention. The results showed that Erastin significantly reversed PS-MP-induced cell proliferation, migration, and invasion, and downregulated the expression of ferroptosis-suppressing proteins such as GPX4. These findings not only confirm the critical involvement of the ferroptosis pathway in the tumor-promoting effects of microplastics but also provide a rationale for the development of ferroptosis-based strategies to counteract environmental carcinogenesis.

From a broader perspective, this study highlights the complex relationship between redox homeostasis in the tumor microenvironment and cancer cell survival strategies. In nasopharyngeal carcinoma—a cancer type highly sensitive to oxidative stress and metabolic reprogramming—the “mild ROS-anti-ferroptosis activation” state induced by PS-MPs may represent a prototypical subclinical carcinogenic process. This phenomenon warrants further validation in clinical specimens and long-term exposure models.

Moreover, this study also suggests that microplastic exposure may act as a contributing factor to tumor immune evasion. Previous studies have shown that ferroptosis can trigger antitumor immune responses by releasing immunogenic molecules [25, 26]. If this process is suppressed by microplastics, it may potentially impair the efficacy of immunotherapy. Therefore, incorporating the interplay between ferroptosis and the immune microenvironment into future research will be instrumental in gaining a more comprehensive understanding of the carcinogenic potential of microplastics.

It should be noted that this study employed 0.5 μm and 1 μm polystyrene microplastics as representative model particles. PS was chosen due to its widespread use in biomedical research, high stability, and uniform particle characteristics, while these two small-size ranges enabled efficient cellular uptake for mechanistic exploration. However, the findings cannot be directly generalized to all types or sizes of microplastics. Future work will extend to other polymers such as polyethylene, polypropylene, or PVC, and to a broader spectrum of particle sizes, in order to validate whether ferroptosis suppression represents a generalizable mechanism of microplastic-driven nasopharyngeal carcinoma progression.

In summary, this study is the first to elucidate, at the cellular, animal, and molecular levels, the mechanism by which microplastics promote the malignant progression of nasopharyngeal carcinoma cells by suppressing ferroptosis via the ROS-GPX4 axis. This finding not only deepens our understanding of the health risks associated with microplastic exposure but also provides a novel theoretical foundation and intervention strategy for research on the tumor microenvironment and ferroptosis-targeted cancer therapy.

## Conclusions

This study reveals a previously unrecognized carcinogenic role of microplastics in nasopharyngeal carcinoma. By driving mitochondrial ROS production and activating the NRF2-SLC7A11/GPX4 antioxidant defense, PS-MPs suppress ferroptosis and thereby accelerate tumor progression. Importantly, pharmacologic ferroptosis reactivation effectively counteracts these malignant effects, underscoring ferroptosis as both a mechanistic link between environmental pollutants and cancer and a tractable therapeutic vulnerability. These findings expand our understanding of environmental carcinogenesis and provide a rationale for developing ferroptosis-based interventions to mitigate cancer risks associated with microplastic exposure.

## Supplementary Information

Below is the link to the electronic supplementary material.

Supplementary Material 1

Supplementary Material 2

Supplementary Material 3

## Data Availability

All data and material will be available upon request to the corresponding author.

## Abbreviations

MPs: Microplastics
PS-MPs: Polystyrene microplastics
CCK-8: Cell Counting Kit-8
EBV: Epstein–Barr virus
ECL: Enhanced chemiluminescence
HE: Hematoxylin and eosin
IC₅₀: Half maximal inhibitory concentration
IHC: Immunohistochemistry
KEGG: Kyoto Encyclopedia of Genes and Genomes
NPC: Nasopharyngeal carcinoma
OD₄₅₀: Optical density at 450 nm
qRT-PCR: Quantitative reverse transcription PCR
RNA-seq: RNA sequencing
ROS: Reactive oxygen species
WB: Western blot

## References

[1] Deng X, Gui Y, Zhao L. The micro(nano)plastics perspective: exploring cancer development and therapy. Mol Cancer. 2025;24:30. 39856719 10.1186/s12943-025-02230-z PMC11761189

[2] Zha H, Li S, Zhuge A, Shen J, Yao Y, Chang K, Li L. Hazard assessment of airborne and foodborne biodegradable polyhydroxyalkanoates microplastics and non-biodegradable polypropylene microplastics. Environ Int. 2025;196:109311. 39892168 10.1016/j.envint.2025.109311

[3] Xiao M, Li X, Zhang X, Duan X, Lin H, Liu S, Sui G. Assessment of cancer-related signaling pathways in responses to polystyrene nanoplastics via a kidney-testis microfluidic platform (KTP). Sci Total Environ. 2023;857:159306. 36216064 10.1016/j.scitotenv.2022.159306

[4] Zhang S, Zhou Y, Liu Z, Wang Y, Zhou X, Chen H, et al. Immunosequencing identifies signatures of T cell responses for early detection of nasopharyngeal carcinoma. Cancer Cell. 2025;43:1423–41. 10.1016/j.ccell.2025.04.009 40345188

[5] Chen YP, Chan ATC, Le QT, Blanchard P, Sun Y, Ma J. Nasopharyngeal carcinoma. Lancet. 2019;394:64–80. 31178151 10.1016/S0140-6736(19)30956-0

[6] Chen L, Liu Y, Li H, Lin S, Wang X, Fang J, Diao X, Wang L, Yang Z, Cai Z. Size-Dependent pulmonary toxicity and Whole-Body distribution of inhaled Micro/Nanoplastic particles in male mice from chronic exposure. Environ Sci Technol. 2025;59:6993–7003. 40181497 10.1021/acs.est.4c14232

[7] Lei G, Zhuang L, Gan B. The roles of ferroptosis in cancer: tumor suppression, tumor microenvironment, and therapeutic interventions. Cancer Cell. 2024;42:513–34. 38593779 10.1016/j.ccell.2024.03.011

[8] Wang Y, Hu M, Cao J, Wang F, Han JR, Wu TW, et al. ACSL4 and polyunsaturated lipids support metastatic extravasation and colonization. Cell. 2025;188:412–e429427. 39591965 10.1016/j.cell.2024.10.047

[9] Ru Q, Li Y, Chen L, Wu Y, Min J, Wang F. Iron homeostasis and ferroptosis in human diseases: mechanisms and therapeutic prospects. Signal Transduct Target Ther. 2024;9:271. 39396974 10.1038/s41392-024-01969-z PMC11486532

[10] Dai E, Chen X, Linkermann A, Jiang X, Kang R, Kagan VE, et al. A guideline on the molecular ecosystem regulating ferroptosis. Nat Cell Biol. 2024;26:1447–57. 38424270 10.1038/s41556-024-01360-8 PMC11650678

[11] Jin X, Tang J, Qiu X, Nie X, Ou S, Wu G, Zhang R, Zhu J. Ferroptosis: emerging mechanisms, biological function, and therapeutic potential in cancer and inflammation. Cell Death Discov. 2024;10:45. 38267442 10.1038/s41420-024-01825-7 PMC10808233

[12] Liu Y, Wan Y, Jiang Y, Zhang L, Cheng W. GPX4: the hub of lipid oxidation, ferroptosis, disease and treatment. Biochim Biophys Acta Rev Cancer. 2023;1878:188890. 37001616 10.1016/j.bbcan.2023.188890

[13] Zou Y, Palte MJ, Deik AA, Li H, Eaton JK, Wang W, et al. A GPX4-dependent cancer cell state underlies the clear-cell morphology and confers sensitivity to ferroptosis. Nat Commun. 2019;10:1617. 30962421 10.1038/s41467-019-09277-9 PMC6453886

[14] Sindhwani S, Syed AM, Ngai J, Kingston BR, Maiorino L, Rothschild J, et al. The entry of nanoparticles into solid tumours. Nat Mater. 2020;19:566–75. 31932672 10.1038/s41563-019-0566-2

[15] Nguyen LNM, Lin ZP, Sindhwani S, MacMillan P, Mladjenovic SM, Stordy B, Ngo W, Chan WCW. The exit of nanoparticles from solid tumours. Nat Mater. 2023;22:1261–72. 37592029 10.1038/s41563-023-01630-0

[16] Li X, Jafari SM, Zhou F, Hong H, Jia X, Mei X, et al. The intracellular fate and transport mechanism of shape, size and rigidity varied nanocarriers for Understanding their oral delivery efficiency. Biomaterials. 2023;294:121995. 36641813 10.1016/j.biomaterials.2023.121995

[17] Yang Z, Su W, Wei X, Qu S, Zhao D, Zhou J, et al. HIF-1alpha drives resistance to ferroptosis in solid tumors by promoting lactate production and activating SLC1A1. Cell Rep. 2023;42:112945. 37542723 10.1016/j.celrep.2023.112945

[18] Zhou Q, Yu H, Chen Y, Ren J, Lu Y, Sun Y. The CRL3(KCTD10) ubiquitin ligase-USP18 axis coordinately regulates cystine uptake and ferroptosis by modulating SLC7A11. Proc Natl Acad Sci U S A. 2024;121:e2320655121. 38959043 10.1073/pnas.2320655121 PMC11252818

[19] Zheng J, Conrad M. The metabolic underpinnings of ferroptosis. Cell Metab. 2020;32:920–37. 33217331 10.1016/j.cmet.2020.10.011

[20] Lan Y, Hu L, Feng X, Wang M, Yuan H, Xu H. Synergistic effect of PS-MPs and cd on male reproductive toxicity: ferroptosis via Keap1-Nrf2 pathway. J Hazard Mater. 2024;461:132584. 37748303 10.1016/j.jhazmat.2023.132584

[21] Huang B, Wang H, Liu S, Hao M, Luo D, Zhou Y, Huang Y, Nian Y, Zhang L, Chu B, Yin C. Palmitoylation-dependent regulation of GPX4 suppresses ferroptosis. Nat Commun. 2025;16:867. 39833225 10.1038/s41467-025-56344-5 PMC11746948

[22] Liang FG, Zandkarimi F, Lee J, Axelrod JL, Pekson R, Yoon Y, Stockwell BR, Kitsis RN. OPA1 promotes ferroptosis by augmenting mitochondrial ROS and suppressing an integrated stress response. Mol Cell. 2024;84:3098–e31143096. 39142278 10.1016/j.molcel.2024.07.020 PMC11373561

[23] Wen D, Li W, Song X, Hu M, Liao Y, Xu D, Deng J, Guo W. NF-kappaB-mediated EAAT3 upregulation in antioxidant defense and ferroptosis sensitivity in lung cancer. Cell Death Dis. 2025;16:124. 39987248 10.1038/s41419-025-07453-y PMC11847022

[24] Glorieux C, Liu S, Trachootham D, Huang P. Targeting ROS in cancer: rationale and strategies. Nat Rev Drug Discov. 2024;23:583–606. 38982305 10.1038/s41573-024-00979-4

[25] Ye P, Wang C, Wen Y, Fang K, Li Q, Zhang X, et al. A positive-feedback loop suppresses TNBC tumour growth by remodeling tumour immune microenvironment and inducing ferroptosis. Biomaterials. 2025;315:122960. 39541840 10.1016/j.biomaterials.2024.122960

[26] Wu B, Yang X, Kong N, Liang J, Li S, Wang H. Engineering modular peptide nanoparticles for Ferroptosis-Enhanced tumor immunotherapy. Angew Chem Int Ed Engl. 2025;64:e202421703. 39721975 10.1002/anie.202421703

