# Supplementary material for: Ferroptosis inhibition via the ROS-GPX4 axis drives microplastic-induced malignant progression of nasopharyngeal carcinoma
Source: J Transl Med. 2025 Dec 22;24:210. doi: 10.1186/s12967-025-07508-w (PMC12903729; doi:10.1186/s12967-025-07508-w)
Supplement: Supplementary file 2 — Supplementary Material 2 [file 12967_2025_7508_MOESM2_ESM.docx]

**Ferroptosis inhibition via the ROS-GPX4 axis drives microplastic-induced malignant progression of nasopharyngeal carcinoma**

**Table S1 qRT-PCR primers**

| **Name** | **sequence** |
| --- | --- |
| GPX4-F | 5'- GCCAGGGAGTAACGAAGAGA -3' |
| GPX4-R | 5'- CAGCCGTTCTTGTCGATGAG -3' |
| SLC7A11-F | 5'- TCCGATCTTTGTTGCCCTCT -3' |
| SLC7A11-R | 5'- GACTGTCGAGGTCTCCAGAG -3' |
| β-actin-F | 5'- TCACCAACTGGGACGACATG -3' |
| β-actin-R | 5'- GTCACCGGAGTCCATCACGAT -3' |
