# Supplementary material for: Ferroptosis inhibition via the ROS-GPX4 axis drives microplastic-induced malignant progression of nasopharyngeal carcinoma
Source: J Transl Med. 2025 Dec 22;24:210. doi: 10.1186/s12967-025-07508-w (PMC12903729; doi:10.1186/s12967-025-07508-w)
Supplement: Supplementary file 3 — Supplementary Material 3 [file 12967_2025_7508_MOESM3_ESM.docx]

**Ferroptosis inhibition via the ROS-GPX4 axis drives microplastic-induced malignant progression of nasopharyngeal carcinoma**


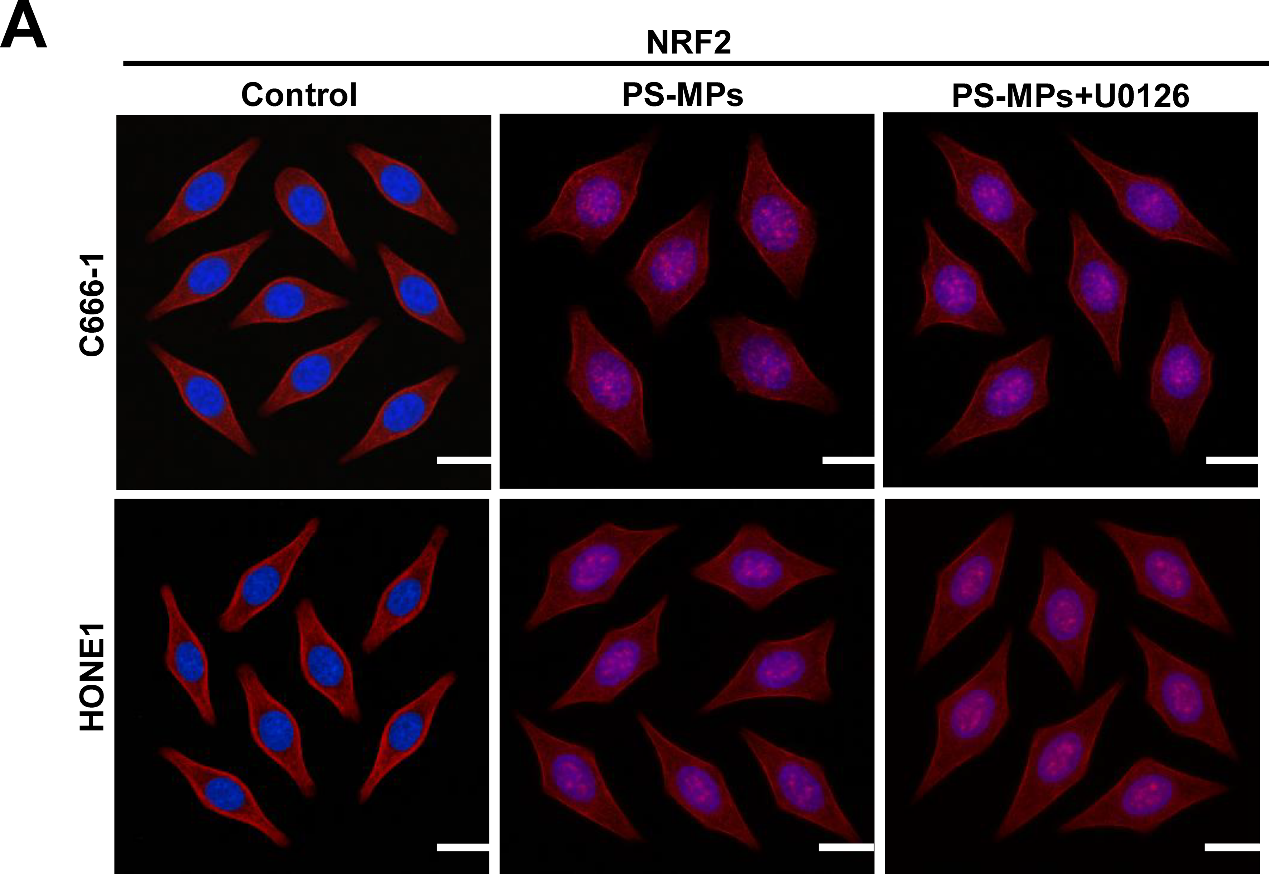


**Fig. S1 PS-MPs induce NRF2 nuclear translocation independently of MAPK signaling. (A)** Inhibition of the MAPK pathway with U0126 does not reverse this effect, as nuclear NRF2 remains elevated compared with control.


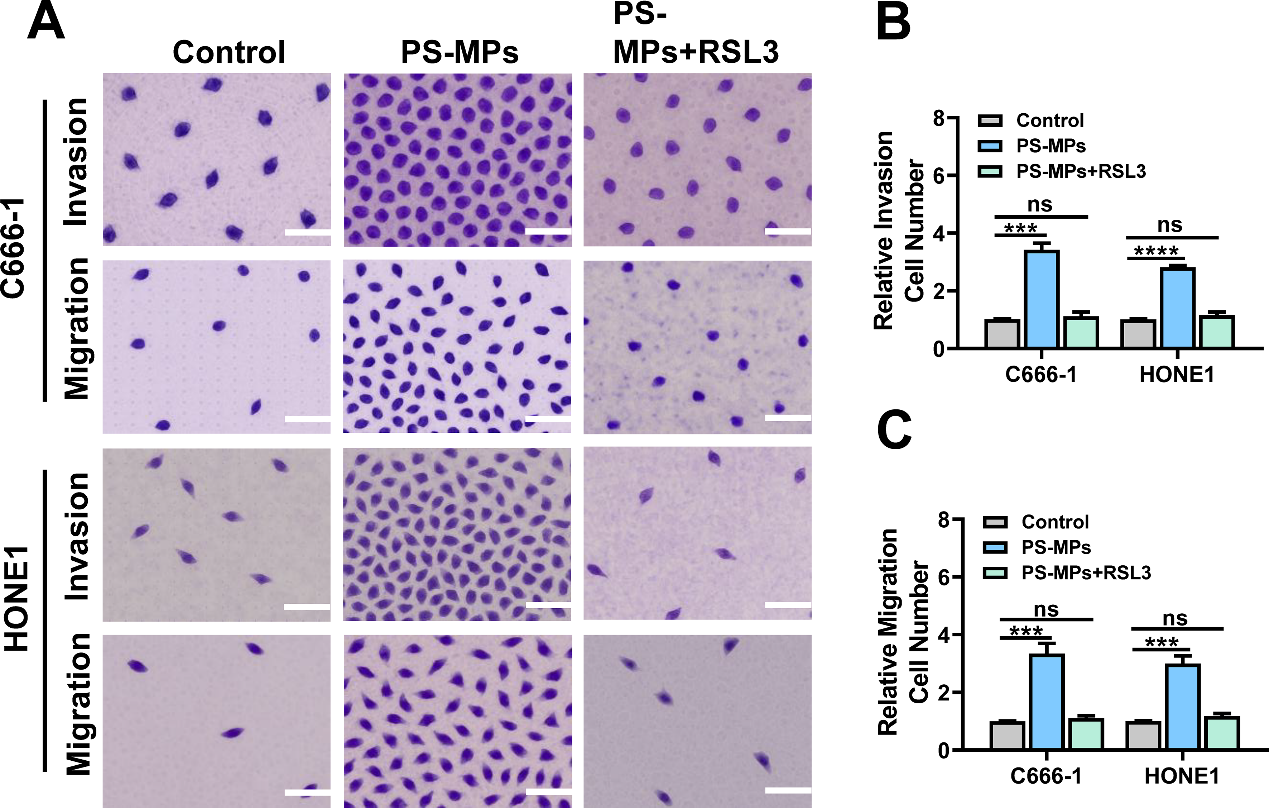


**Fig. S2 RSL3 reverses PS-MPs-induced enhancement of migration and invasion in NPC cells. (A-C)** The intervention with the GPX4 inhibitor RSL3 was found to significantly attenuate the migration and invasion abilities of NPC cells induced by PS-MPs.
